# Early transcriptional response to aminoglycoside antibiotic suggests alternate pathways leading to apoptosis in sensory hair cells in the mouse inner ear

**DOI:** 10.3389/fncel.2015.00190

**Published:** 2015-05-21

**Authors:** Litao Tao, Neil Segil

**Affiliations:** ^1^Genetic, Molecular and Cellular Biology Program, University of Southern CaliforniaLos Angeles, CA, USA; ^2^Department of Stem Cell Biology and Regenerative Medicine, University of Southern CaliforniaLos Angeles, CA, USA; ^3^Department of Otolaryngology, University of Southern CaliforniaLos Angeles, CA, USA

**Keywords:** sensory hair cells, aminoglycoside antibiotics, gentamicin, ototoxicity, RNA sequencing, differential gene expression

## Abstract

Aminoglycoside antibiotics are “the drug of choice” for treating many bacterial infections, but their administration results in hearing loss in up to one fourth of the patients who receive them. Several biochemical pathways have been implicated in aminoglycoside antibiotic ototoxicity; however, little is known about how hair cells respond to aminoglycoside antibiotics at the transcriptome level. Here we have investigated the genome-wide response to the aminoglycoside antibiotic gentamicin. Using organotypic cultures of the perinatal organ of Corti, we performed RNA sequencing using cDNA libraries obtained from FACS-purified hair cells. Within 3 h of gentamicin treatment, the messenger RNA level of more than three thousand genes in hair cells changed significantly. Bioinformatic analysis of these changes highlighted several known signal transduction pathways, including the JNK pathway and the NF-κB pathway, in addition to genes involved in the stress response, apoptosis, cell cycle control, and DNA damage repair. In contrast, only 698 genes, mainly involved in cell cycle and metabolite biosynthetic processes, were significantly affected in the non-hair cell population. The gene expression profiles of hair cells in response to gentamicin share a considerable similarity with those previously observed in gentamicin-induced nephrotoxicity. Our findings suggest that previously observed early responses to gentamicin in hair cells in specific signaling pathways are reflected in changes in gene expression. Additionally, the observed changes in gene expression of cell cycle regulatory genes indicate a disruption of the postmitotic state, which may suggest an alternate pathway regulating gentamicin-induced apoptotic hair cell death. This work provides a more comprehensive view of aminoglycoside antibiotic ototoxicity, and thus contributes to identifying potential pathways or therapeutic targets to alleviate this important side effect of aminoglycoside antibiotics.

## Introduction

Ototoxicity is a well-known side effect limiting the use of aminoglycoside antibiotics, with reported incidence of hearing loss between 2% and 25% of treated patients (Huth et al., 2011). Several biochemical mechanisms of aminoglycoside ototoxicity have been investigated, including production of reactive oxygen species (ROS) (Forge and Schacht, 2000), disruption of intracellular calcium storage (Matsui et al., 2004), and inhibition of cytoplasmic protein synthesis (Francis et al., 2013). Through biochemical assays, some pathways have also been identified as signaling pathways mediating aminoglycoside-induced hair cell death, such as the pro-apoptotic JNK pathway (Ylikoski et al., 2002) and the protective NF-κB pathway (Jiang et al., 2005). Based on observations made in organ cultures, a timeline of critical signaling events has been established. ROS production is induced shortly after aminoglycoside administration; the JNK pathway responds to drug treatment within 3 h; the intracellular calcium level is increased dramatically at around 4 h; cytochrome C is released into cytoplasm at around 12 h and apoptosis execution steps occur at around 18 h (Matsui et al., 2004).

The methods utilizing low-throughput biochemical assays have been instrumental in dissecting some of the sequelae of antibiotic treatment of hair cells. However, cells are complex systems that integrate input from multiple mechanisms and respond by adjusting multiple pathways simultaneously. High-throughput methods may help us gain a better understanding of the ototoxicity associated with aminoglycosides by providing a complex view of the response; among them, DNA microarray and antibody array experiments have been performed to profile the changes in the cochlea after ototoxic drug treatment at the transcriptional level and the protein level, respectively (Nagy et al., 2004; Jamesdaniel et al., 2008). However, whole cochlea samples were used in those studies, which consisted largely of many different non-hair cell populations in addition to hair cells, and the heterogeneity of the samples limited the interpretation of the results due to a low signal to noise ratio.

Here we have utilized RNA sequencing to investigate the early gene expression changes in hair cells induced by the aminoglycoside antibiotic gentamicin. Compared to microarray analysis, RNA sequencing is more accurate for analyzing expression levels, more informative for pathway analysis, and more powerful in exploiting unannotated genes (Wang et al., 2009). We made use of the Atoh1-GFP transgenic mice, whose fluorescent reporter under the control of the Atoh1 enhancer faithfully identifies hair cells (Lumpkin et al., 2003), to purify GFP+ hair cells by fluorescence-activated cell sorting (FACS) after a 3 h gentamicin treatment for a direct analysis of gene expression changes by RNA sequencing. We chose the 3 h time point because it was previously shown that signaling pathways have initiated a response to the drug, but the cell death process did not appear to be initiated (Matsui et al., 2004). We present analyses of the gene changes associated with several major pathways known to be dysregulated in response to aminoglycoside antibiotics.

Genome wide, 3709 genes, including those involved in a variety of pathways and cellular processes, were found to change significantly in hair cells after gentamicin treatment. Genes involved in the JNK pathway and the NF-κB pathway were differentially expressed in gentamicin-treated hair cells, which is consistent with previous findings that these pathways are important for aminoglycoside ototoxicity (Ylikoski et al., 2002; Jiang et al., 2005). Surprisingly, we did not observe a strong stress response at the transcriptional level at this early time point. Comparison of gentamicin-induced gene expression changes in hair cells and data from other cell lines and tissues, revealed a similarity between aminoglycoside-induced hair cell death and neuronal cell death, as well as a resemblance between aminoglycoside ototoxicity and nephrotoxicity. Additionally, genes involved in the cell cycle and DNA damage repair were also strongly affected by gentamicin at the transcriptional level, indicating a gentamicin-induced disruption of the postmitotic state, which may underlie an alternate pathway leading to hair cell death. In contrast, significantly fewer genes (698 genes) with altered expression after gentamicin treatment were present in the non-hair cell population at this time. Interestingly, expression of genes involved in cell cycle and metabolite biosynthesis pathways were significantly affected in this population. Analyses of our RNA sequencing data indicate that prior to severe oxidative stress, multiple events, including previously implicated activation of the JNK and the NF-κB pathways, and newly hypothesized disruption of the postmitotic state, occur in hair cells in response to aminoglycoside antibiotic treatment. Our work provides a comprehensive view of aminoglycoside ototoxicity, and our transcriptome data could be used to analyze other potential signaling pathways or biological processes mediating aminoglycoside-induced hair cell death which are not covered here.

## Materials and methods

### Organotypic culture, drug treatment, and FACS sorting

Atoh1-GFP transgenic mice on CD-1 background (Lumpkin et al., 2003; available in the Jackson Laboratory but in different genomic background: B6.129S-Atoh1^tm4.1Hzo^/J) were used for this study. All animal procedures and usage were approved by the IACUC committee in House Research Institute. Inner ears were collected from postnatal day 1 (P1) animals under sterile conditions and then transferred into ice cold Ca^2+^- and Mg^2+^-free PBS (Invitrogen), in which cochlea were further dissected under the microscope (Chen et al., 2002). The cochleae were mounted on polycarbonate membranes (13 mm diameter, 1.0 μm pore size, SPI supplies) floating on DMEM/F12 medium (Invitrogen) supplemented with 1% N2 (Gibco) and 100 U/ml penicillin (Sigma). Organ cultures were maintained under low oxygen conditions (37°C, 5% CO_2_ and 5% O_2_) overnight, and treated with gentamicin the next day.

To trace the accumulation of gentamicin in hair cells, Texas Red conjugated gentamicin (GTTR) was prepared as described in the literature (Steyger et al., 2003). 4.4 ml of gentamicin sulfate (Sigma; 50 mg/ml in K_2_CO_3_, pH 9) was mixed with 0.6 ml succinimidyl ester of Texas Red (Invitrogen; 2 mg/ml in dimethyl formamide), and then agitated overnight to produce a mixture of gentamicin and GTTR with an approximate 300:1 molar ratio. This GTTR mixture solution was diluted 1:100 with culture medium for treatment of organotypic cultures. After 30 min incubation, organs were fixed with 4% paraformaldehyde, cryosectioned and photographed by fluorescence microscopy.

**Figure 1:**
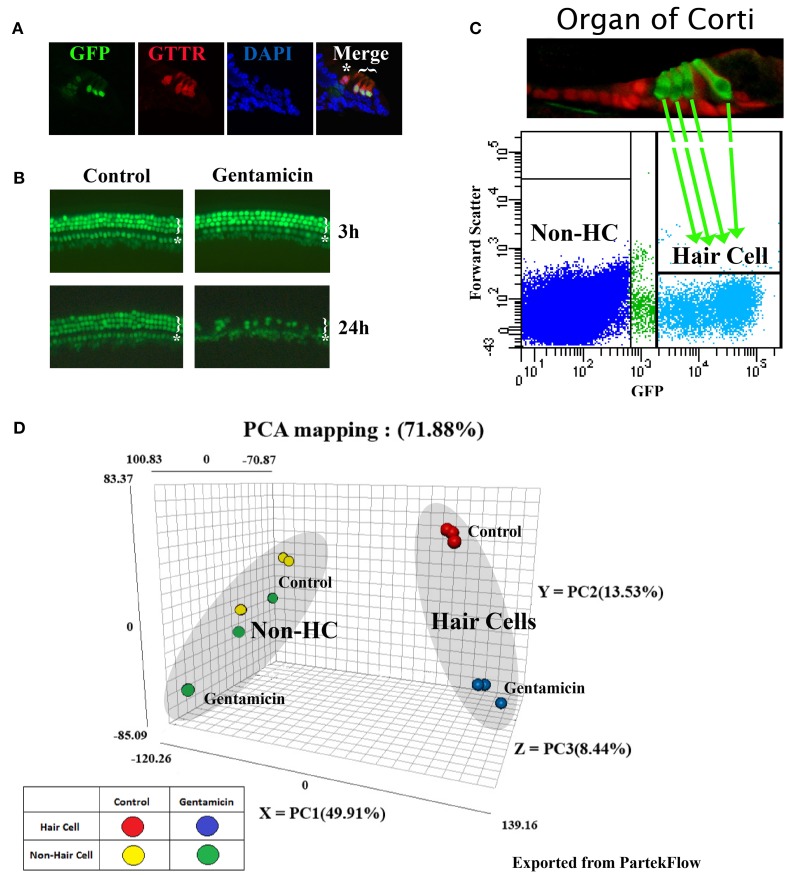
**Gentamicin-induced hair cell loss, and principal component analysis (PCA) of RNA sequencing datasets. (A)** Gentamicin accumulates preferentially in hair cells in *in vitro* organ of Corti culture. Cross sections through cochlear explants from P1, Atoh1-GFP mice (Lumpkin et al., 2003), treated for 30 min with Texas Red conjugated gentamicin (GTTR). Star indicates the inner hair cell, and bracket indicates three outer hair cells. Cells with weak GFP under the inner hair cell are inner phalangeal cells. **(B)** Atoh1-GFP fluorescence in untreated and 0.5 mM gentamicin-treated organs at 3 and 24 h. There was no detectable hair cell loss at 3 h, but severe hair cell damage caused by gentamicin at 24 h. Star indicates a single row of inner hair cells and bracket indicates three partial rows of outer hair cells. **(C)** Scatter plot of Atoh1-GFP hair cell purification by FACS shows gate settings and diagram shows a P1 organ of Corti indicating hair cells (green) and supporting cells (red) (Chen and Segil, 1999). **(D)** PCA map showing the three most significant variances among samples. 78.88% of variance in the combined dataset is captured in the analysis; (49.91% in PC1-X axis, 13.53% in PC2-Y axis, and 8.44% on PC3-Z axis). Each dot represents one biological replicate.

To confirm the level of hair cell death induced by gentamicin, organ cultures were treated with 0.5 mM gentamicin for 3 h, and then gentamicin was washed out and replaced with fresh medium. GFP positive hair cells were photographed at 3 and 24 h timepoints (Figure 1B).

To purify hair cells for RNAseq, organs were digested with 0.05% Trypsin (Invitrogen) and 1 mg/ml Collagenase (Worthington) in PBS at 37°C for 8 min, then incubated with 10% FBS (Life Technologies) in PBS to stop enzymatic digestion. To make single cell suspensions, organs were triturated with a P200 pipette 300 times. The suspension was passed through a cell strainer (40 μm, BD Biosciences) before FACS purification. GFP-positive hair cells, as well as the GFP-negative non-hair cell population (non-hair cell cochlear epithelial cells included Deiters' cells, pillar cells, Hensen cells, cells in the GER, cells in the LER, and other cells constituting surrounding tissues) were purified on a BD FACS Aria II with a 100 μ nozzle. Cells with low-levels of GFP were excluded by stringent gating during FACS purification (Figure 1C). Quality control by FACS-resort, and by immunofluorescence for a hair cell marker (MyosinVI), indicated >95% purity. Sorted cells were collected directly into RNA lysis buffer (Zymo). At least 50,000 cells were collected for each sample, and three replicates were prepared for each condition.

### RNA sequencing, reads alignment, PCA and differential gene expression

RNA was extracted from samples using the Zymo Quick-RNA Microprep kit, and then processed for library construction, using the Illumina True-Seq mRNA-seq kit. Six samples were bar-coded, combined into one lane, and sequenced by Illumina Hi-Seq 2000 for single-end 50 cycles (50 bp reads). More than 30 million reads were obtained for each replicate. The reads were trimmed on both ends (quality score ≥25) and aligned against the mouse genome assembly mm10 using TopHat 2 in PartekFlow (Partek Inc.). Normalized read score for each gene was calculated considering total read numbers and gene length (reads per kilobase of transcript per million reads mapped, RPKM). Principal component analysis (PCA) was conducted in PartekFlow based on normalized read numbers for individual genes in each replicate. Differential gene expression was assessed by the embedded gene specific analysis (GSA) module in PartekFlow.

RNA sequence data was deposited into NCBI GEO database (GSE66775).

### IPA analysis

Differential gene expression datasets, including gene symbols, fold changes, *p*-values and total numbers of raw reads, were prepared for Ingenuity pathway analysis software (IPA, Version 21901358, Qiagen Inc.). The gene expression dataset from untreated and gentamicin-treated hair cells, and from untreated and treated non-hair cell samples, were analyzed by the IPA software. A filter of *p*-value less than 0.01, fold change greater than 1.2 (or less than −1.2), and total number of raw reads greater than 100 was applied, and core pathway analysis was conducted for each dataset. Canonical pathway analysis, diseases and function analysis, upstream regulator analysis, and network analysis were included in the core analysis with default settings.

### Q-PCR validation

The cDNA libraries for Q-PCR were made by qScript reverse transcriptase supermix (Quanta Biosciences) using RNA extracted from FACS purified cells as template. *Rpl19* was used as internal control for normalization. For validation purpose, four independent biological replicates were collected and analyzed by Q-PCR. Genes were chosen arbitrarily among the list of gentamicin-induced genes in hair cells. SYBR-Green (Applied Biosystems) was used to detect amplified double strand DNA on ViiA 7 machine (Applied Biosystems). Primer pairs used for Q-PCR were listed below. *Rpl19* forward 5′-GGTCTGGTTGGATCCCAATG-3′, reverse 5′-CCCGGGAATGGACAGTCA-3′. *Atf2* forward 5′-CCGTTGCTATTCCTGCATCAA-3′, reverse 5′-TTGCTTCTGACTGGACTGGTT-3′. *Mapk8* forward 5′-AGCAGAAGCAAACGTGACAAC-3′, reverse 5′-GCTGCACACACTATTCCTTGAG-3′. *Jun* forward 5′-CCTTCTACGACGATGCCCTC-3′, reverse 5′-GGTTCAAGGTCATGCTCTGTTT-3′. *Nfkb1* forward 5′-ATGGCAGACGATGATCCCTAC-3′, reverse 5′-TGTTGACAGTGGTATTTCTGGTG-3′. *Nfkbib* forward 5′-GCGGATGCCGATGAATGGT-3′, reverse 5′-TGACGTAGCCAAAGACTAAGGG-3′. *Chuk* forward 5′-GTCAGGACCGTGTTCTCAAGG-3′, reverse 5′-GCTTCTTTGATGTTACTGAGGGC-3′. *Gadd45g* forward 5′-GGGAAAGCACTGCACGAACT-3′, reverse 5′-AGCACGCAAAAGGTCACATTG-3′. *Klf11* forward 5′-CATGGACATTTGTGAGTCGATCC-3′, reverse 5′-CCTTTGGTAGATCAGGTGCAG-3′. *Ddit3* forward 5′-CTGGAAGCCTGGTATGAGGAT-3′, reverse 5′-CAGGGTCAAGAGTAGTGAAGGT-3′. *Bnip1* forward 5′-AGGCTATGCAGACTCTAGTCAG-3′, reverse 5′-CAGTTCTCGGCGGTTGTACT-3′. *Casp3* forward 5′-ATGGAGAACAACAAAACCTCAGT-3′, reverse 5′-TTGCTCCCATGTATGGTCTTTAC-3′. *Ccar1* forward 5′-AGATGAGTATGACCCAATGGAGG-3′, reverse 5′-CCTTGCAGTACCGGCTGAC-3′. *Ccnb2* forward 5′-GCCAAGAGCCATGTGACTATC-3′, reverse 5′-CAGAGCTGGTACTTTGGTGTTC-3′. *Ccne2* forward 5′-ATGTCAAGACGCAGCCGTTTA-3′, reverse 5′-GCTGATTCCTCCAGACAGTACA-3′. *Ercc8* forward 5′-GAGGAAGATGAAGCTATGGAA-3′, reverse 5′-CTTCAGGGGTTTCTCTTTGTC-3′. *Rad52* forward 5′-CTTTGTTGGTGGGAAGTCTGT-3′, reverse 5′-CGGCTGCTAATGTACTCTGGAC-3′. *Apex2* forward 5′-GGATGGATGGCTTGCTCAGTA-3′, reverse 5′-ACTTCAGGGAGTAAGAAGGAGG-3′. *Alkbh3* forward 5′-GAGCCAGTCTGCTACTCAGC-3′, reverse 5′- AACACAAATTGTCGGTCACATTG-3′.

## Results

**Figure 2:**
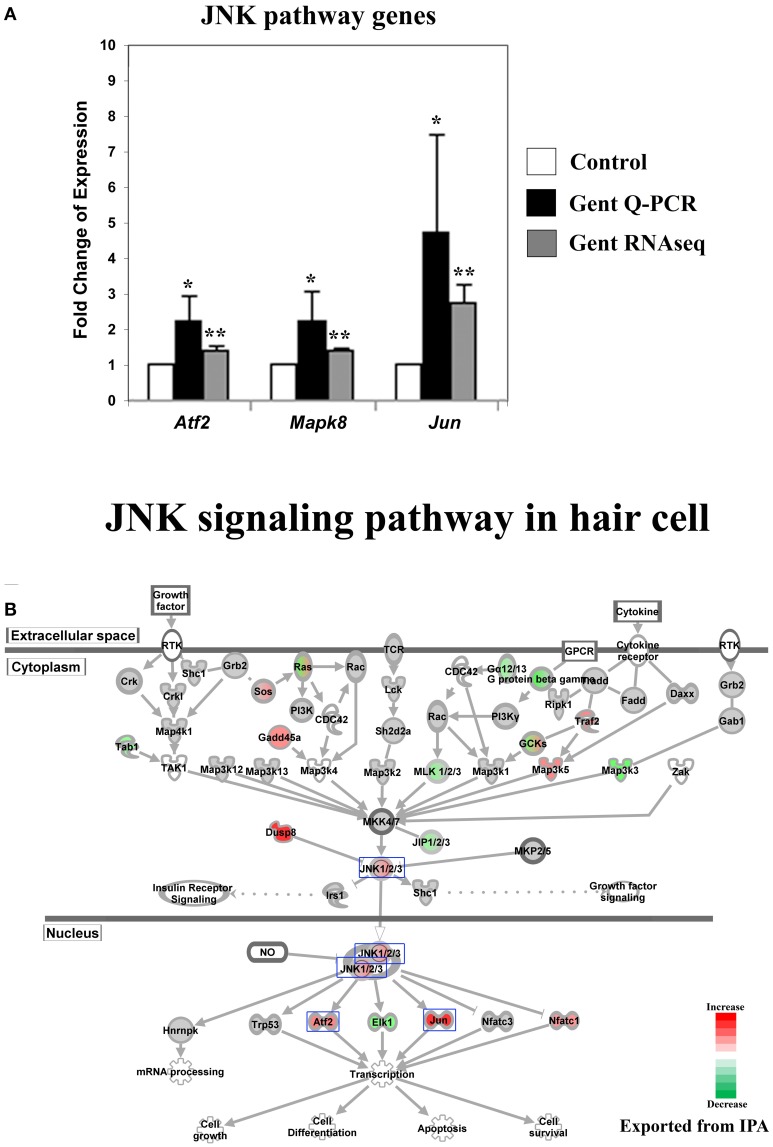
**Gentamicin-induced changes in expression of genes in the Jun-kinase (JNK) pathway. (A)** Q-PCR results validating increased messenger RNA levels in hair cells after gentamicin treatment. Error bar, standard deviation: ^*^p < 0.05; ^**^p < 0.01. **(B)** The JNK signaling pathway with color-coded expression changes indicating significant transcriptional response to gentamicin. Only genes with significant expression changes are colored. Red, increased expression; green, decreased expression relative to control; half red-half green, indicates nodes with multiple genes with some genes upregulated and some genes downregulated. Genes validated and discussed in the text are boxed in the corresponding protein pathway diagrams.

**Figure 3:**
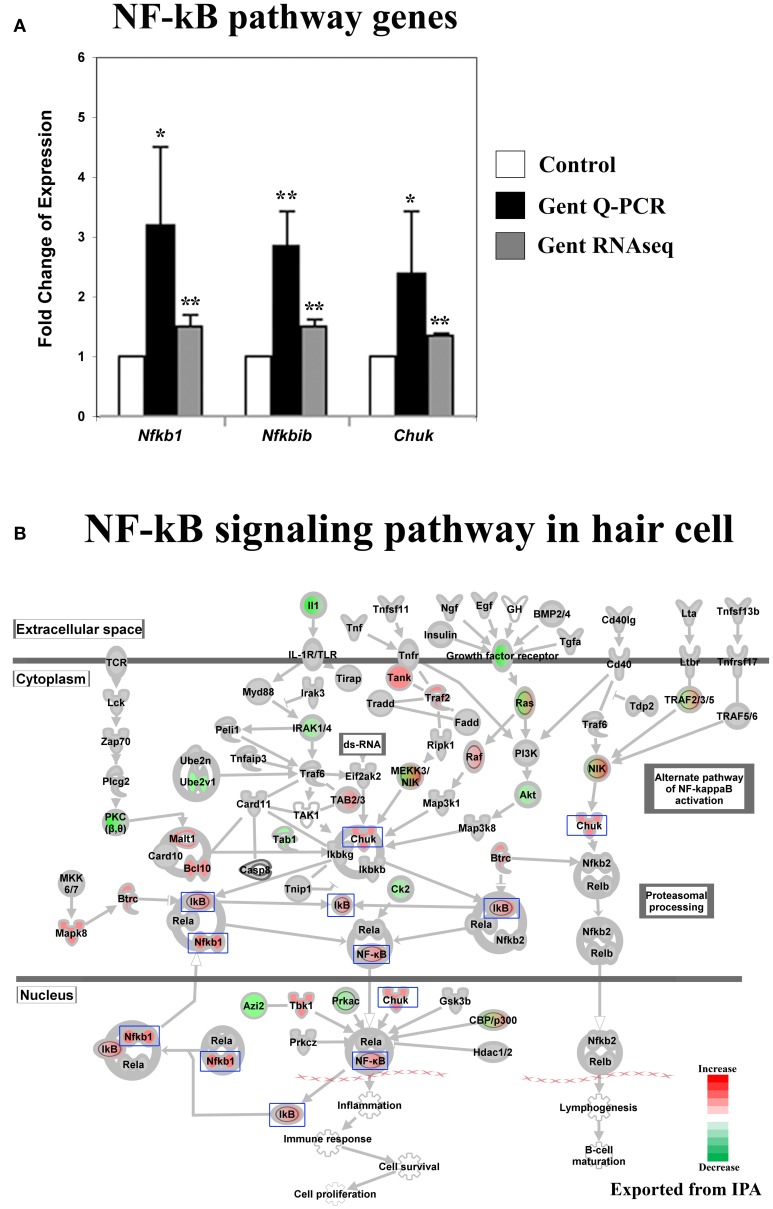
**Gentamicin-induced changes in expression of genes in the NF-κB pathway. (A)** Q-PCR results validating increased messenger RNA levels in hair cells after gentamicin treatment. Error bar, standard deviation: ^*^p < 0.05; ^**^p < 0.01. **(B)** NF-κB pathway with color-coded expression changes suggesting early transcriptional response to gentamicin. Only genes with significant expression changes are colored. Red, increased expression; green, decreased expression relative to control; half red-half green, symbols for nodes consisting of multiple genes with some genes upregulated and some genes downregulated. Genes validated and discussed in the text are boxed in the corresponding protein pathway diagrams.

**Figure 4:**
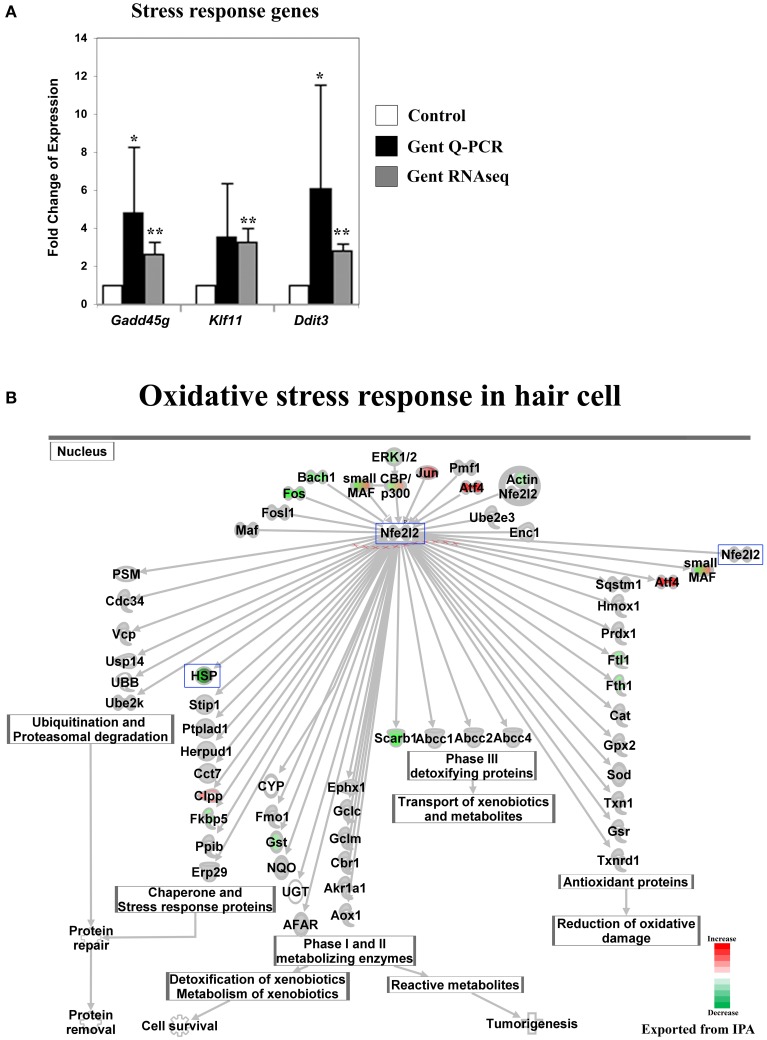
**Gentamicin-induced changes in expression of stress-response genes. (A)** Q-PCR results validating increased messenger RNA levels in hair cells after gentamicin treatment. Error bar, standard deviation: ^*^p < 0.1; ^**^p < 0.01. **(B)** NRF2-mediated oxidative stress response network with color-coded expression shifts showing the absence of significant induction of oxidative stress response at the transcriptional level. Only genes with significant expression changes are color labeled. Red, increased expression; green, decreased expression relative to control; half red-half green, indicates nodes with multiple genes with some genes upregulated and some genes downregulated. Genes validated and discussed in the text are boxed in the corresponding protein pathway diagrams.

**Figure 5:**
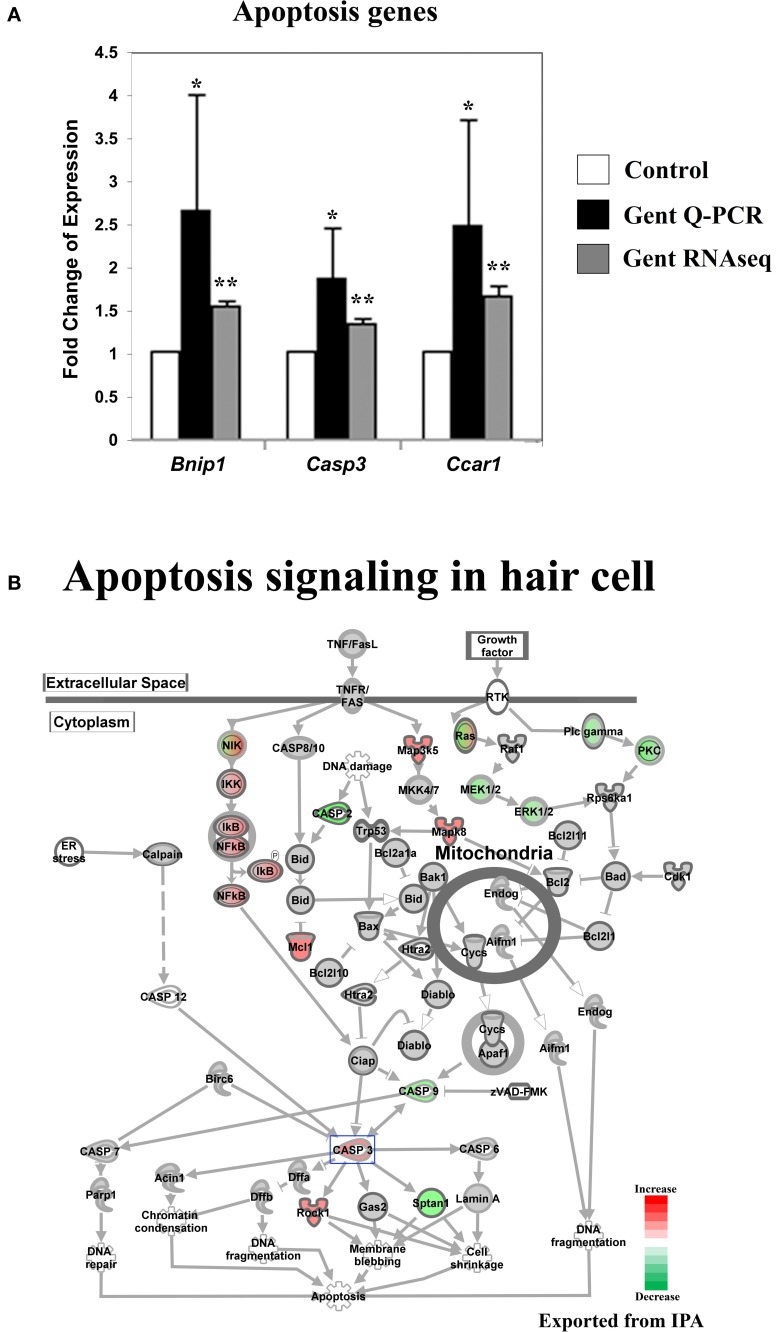
**Gentamicin-induced changes in expression of genes involved in apoptosis. (A)** Q-PCR results validating increased messenger RNA levels in hair cells after gentamicin treatment. Error bar, standard deviation: ^*^p < 0.05; ^**^p < 0.01. **(B)** The apoptosis gene network with color-coded expression changes showing the absence of significant changes for apoptosis initiation and execution genes at transcriptional level. Only genes with significant expression changes are colored. Red, increased expression; green, decreased expression relative to control; half red-half green, indicates nodes with multiple genes with some genes upregulated and some genes downregulated. Genes validated and discussed in the text are boxed in the corresponding protein pathway diagrams.

**Figure 6:**
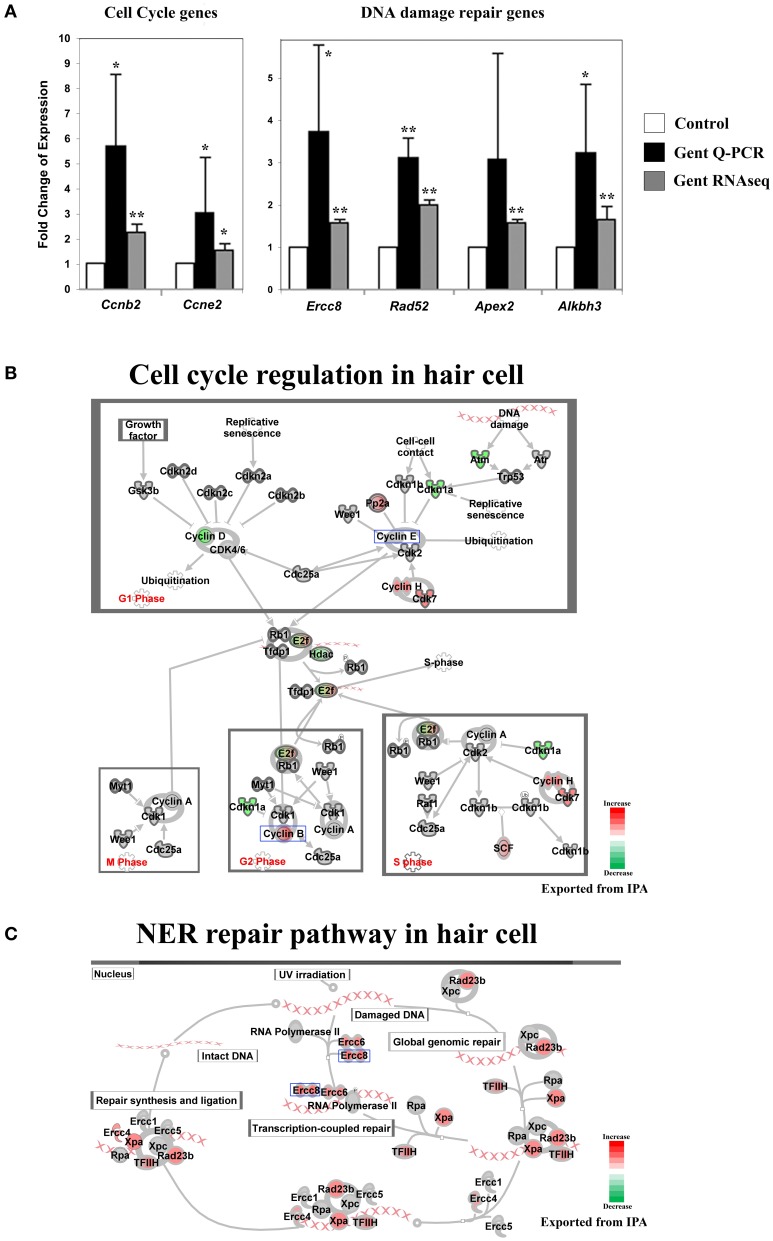
**Gentamicin-induced changes in expression of genes involved in cell cycle and DNA damage repair. (A)** Q-PCR results validating increased messenger RNA levels of selected genes in hair cells after gentamicin treatment. These results confirm the up-regulation of cell cycle and DNA damage repair genes in gentamicin-treated hair cells. Error bar, standard deviation: ^*^p < 0.05; ^**^p < 0.01. **(B)** Cell cycle regulation network with color-coded expression changes suggesting affected cell cycle gene regulation by gentamicin. **(C)** Nucleotide excision repair (NER) network with color-coded expression changes showing significant induction of NER genes. Only genes with significant expression changes are colored. Red, increased expression; green, decreased expression relative to control; half red-half green, indicates nodes with multiple genes with some genes upregulated and some genes downregulated. Genes validated and discussed in the text are boxed in the corresponding protein pathway diagrams.

Perinatal cochleae from Atoh1-GFP transgenic mice, cultured and treated with gentamicin, show that gentamicin accumulated specifically in hair cells, as indicated by the uptake of Texas-Red conjugated gentamicin (Figure 1A), and 91% (SD ± 7%; *n* = 3) of outer hair cells were killed by gentamicin at 24 h (Figure 1B). To investigate the early transcriptional response of hair cells to gentamicin, cultured cochleae were treated with gentamicin for 3 h, and immediately dissociated and FACS-sorted to obtain purified hair cell and non-hair cell samples (Figure 1C) for RNA sequencing. Since there is a low level of misexpression of GFP in inner phalangeal cells and probably in border cells, cells with low-level GFP expression were excluded by stringently gating GFP during FACS purification to minimize possible contamination. We validated the purity of the hair cell population by immunostaining, as more than 95% of purified GFP-positive cells stained positive for the hair cell marker MyoVI (data not shown). In addition, known hair cell specific genes, such as *Atoh1, Pou4f3, Myo6*, and *Myo7a*, have much greater normalized read numbers in hair cell samples than that in non-hair cell samples (Table S1), suggesting the high purity of our hair cell sample. The reliability of the RNA sequencing data was verified by Q-PCR analysis with primers specific for selected genes (Figures 2A, 3A, 4A, 5A, 6A).

Expression of 23420 genes was quantified and analyzed with the Negative Binomial, Poisson or Lognormal Linear distribution model (gene specific analysis algorithm provided by PartekFlow) (Table S1). After 3 h of gentamicin treatment, the mRNA level of 3709 genes in hair cells was significantly altered, with 1917 genes found to be up-regulated and 1792 genes down-regulated (*p*-value < 0.01, fold change > 1.2 or < −1.2, and raw reads ≥100). In contrast, significantly fewer genes (698) were found to be affected with the same criteria in the non-hair cell population, with 436 genes up-regulated and 262 genes down-regulated.

### Principal components analysis

Principal components analysis (PCA) is a method for analyzing the variance in high dimensional datasets, and to present variance in a few dimensions for easier interpretation (Ringnér, 2008). The PCA analysis of our RNA sequencing data shows that 71.88% variance in the dataset was captured within the three most significant principal components (PCs): 49.91% in PC1 (X axis), 13.53% in PC2 (Y axis), and 8.44% in PC3 (Z axis) (Figure 1D). These PCs correspond to different cell types and different treatment conditions.

In the PCA map, each biological replicate of different experimental groups is represented by one dot (Figure 1D). PCA analysis indicates that the variance among the replicates of FACS-purified hair cells is low. Three untreated hair cell replicates cluster tightly in the PCA map (red dots), as do three gentamicin-treated hair cell replicates (blue dots) (Figure 1D). This tight clustering indicates low statistical variance between replicates, and likely stems from the low cellular heterogeneity among FACS-purified replicates. In contrast, both treated (green dots) and untreated (yellow dots) samples from the non-hair cell replicates, which include all other cells in our dissected epithelial preparations, are scattered in the PCA map (Figure 1D), indicating the higher statistical variance among replicates of the non-hair cell samples. Hair cell samples and the non-hair cell samples (Figure 1D, red vs. yellow) lie at the two ends of the most significant variance axis (PC1, X axis), suggesting that the hair cell gene expression profile is significantly different from the non-hair cell population. Along the second variance axis (PC2, Y axis), gentamicin-treated hair cell samples are separated from untreated hair cell samples (Figure 1D, blue vs. red), indicating the significant gene expression shift in hair cells caused by gentamicin treatment at the 3 h time point. In contrast, the non-hair cell samples are not separated by gentamicin treatment (Figure 1D, green vs. yellow), suggesting that the gentamicin-induced gene expression shift in the non-hair cell population was not as consistent as the one in hair cells. Together, the PCA results suggest that gentamicin treatment induced significant and consistent gene expression changes in hair cells.

### Signaling pathways and biological processes affected by gentamicin in hair cells

Gene expression in hair cells was affected significantly by gentamicin treatment at 3 h, as shown by the large number of deregulated genes and by the PCA results. To investigate the underlying biological pathways affected in hair cells upon gentamicin treatment, IPA software (Qiagen) was used to analyze affected signaling pathways after gentamicin treatment, and to compare our hair cell datasets with datasets derived from other cell lines and tissues. Pathways affected by gentamicin treatment at 3 h are listed in Table S2. Below we verify and discuss several of these pathways that have been reported to be important in aminoglycoside induced hair cell death, as well as proposing that elements of the cell cycle may provide an additional pathway, signaling apoptosis in hair cells.

#### Expression of JNK pathway-related genes was significantly affected by gentamicin

The JNK pathway has been strongly implicated in aminoglycoside-induced hair cell death. After aminoglycoside treatment, phosphorylation of JNK and c-Jun was observed in hair cells (Ylikoski et al., 2002), indicating activation of the JNK pathway. Furthermore, inhibition of the JNK pathway by pharmaceutical inhibitors has been shown to protect hair cells from aminoglycoside ototoxicity (Ylikoski et al., 2002), suggesting that JNK activation may be involved in mediating aminoglycoside-induced hair cell death.

Consistent with these findings, we observed changes in the expression of genes involved in JNK signaling pathways upon gentamicin treatment. We verified the expression changes of three genes in this pathway by Q-PCR, namely *Atf2, Mapk8* (*Jnk1*), and *Jun* (*c-Jun*) (Figure 2A). IPA pathway analysis revealed that the expression of JNK pathway-related genes was significantly affected in gentamicin-treated hair cells (*p* = 0.000001). As shown in Figure 2B (color coded, see legend), *Mapk8* (*Jnk1*) itself, in addition to several upstream and downstream factors including *Jun, Atf2, Nfatc1*, and *Elk1*, were changed at the transcriptional level in gentamicin-treated hair cells. Interestingly, the induction of *Jun* expression is regulated by phosphorylation of Jun itself, in an autoregulatory loop, by JNK (Angel et al., 1988); thus the increased mRNA expression of *Jun* indicates the likely activation of the JNK pathway. Transcriptional changes in other targets of Jun regulation are summarized in Table S3. The pattern of changed expression of JNK pathway-related genes is consistent with the timeline of critical signaling events for aminoglycoside-induced hair cell death, indicating that the JNK pathway is one of the early response pathways that are activated in hair cells after aminoglycoside treatment (Matsui et al., 2004).

#### Genes involved in the NF-κB pathway were expressed differentially after gentamicin treatment

Activation of the NF-κB pathway has been implicated as protective against aminoglycoside ototoxicity in several biochemical assays (Jiang et al., 2005). Aminoglycoside treatment has been shown to increase DNA binding activity of NF-κB, indicating activation of the NF-κB pathway by aminoglycoside antibiotics (Jiang et al., 2005). Additionally, reagents promoting NF-κB translocation into nuclei reportedly protect hair cells from aminoglycoside ototoxicity (Jiang et al., 2005).

Our RNA sequencing data show that the expression of genes involved in the NF-κB pathway was altered in hair cells after gentamicin treatment. Gentamicin-induced expression changes of *Nfkb1* (*p50*), *Nfkbib* (*Ikbb*), and *Chuk*(*Ikk1*) were verified by Q-PCR (Figure 3A). Fold changes based on RNA sequencing were smaller but remained highly significant (*p* < 0.01). In IPA pathway analysis, the NF-κB pathway was identified as a significantly affected pathway in gentamicin-treated hair cells (*p* = 0.00004). Expression changes of genes involved in the NF-κB pathway are shown in Figure 3B. Among these genes, *Nfkb1* and *Nfkbib* have been identified as target genes of NF-κB (Ten et al., 1992; Schreiber et al., 2006), and their increased expression strongly suggests the activation of the NF-κB pathway in gentamicin-treated hair cells. Transcriptional changes in other NF-κB targets are summarized in Table S3. Our expression data indicate that the NF-κB pathway, like the JNK pathway, is an early response pathway that is activated in hair cells by aminoglycosides.

#### No significant stress response was observed at the transcriptional level for NRF2 target genes at the 3 h time point

The prevailing hypothesis of aminoglycoside ototoxicity holds that aminoglycoside-induced ROS production causes oxidative stress, which triggers apoptosis in hair cells. Cellular ROS levels are reported to increase within minutes of aminoglycoside administration *in vitro* (Hirose et al., 1997), and antioxidant reagents are shown to protect hair cells from aminoglyocside ototoxicity (Schacht, 1999). In addition, the oxidative stress response mediated by the transcription factor NRF2 (Nfe2l2) has been implicated in aminoglycoside ototoxicity (Hoshino et al., 2011). Therefore, a response to oxidative stress at the transcriptional level was expected. Surprisingly, although some genes upstream of the Nrf2-dependent oxidative stress pathway were upregulated in response to gentamicin at 3 h, the transcriptional key regulator Nrf2 (Nfe2l2), was not affected, nor were its well-studied transcriptional targets (Ma, 2013) (Figure 4B and Table S3). We interpret this to mean that at this early time point, the Nrf2 oxidative stress pathway has not been fully activated. One explanation for this might be that at the 3 h time point, aminoglycoside-induced ROS production had not increased to a level sufficient to overwhelm the highly-developed antioxidant system in hair cells, and this hypothesis is supported by the observation that after the initial disruption, a new balanced redox state indicated by stable NAD(P)H fluorescence is reached within 15 min post-gentamicin treatment (Schafer and Buettner, 2001; Tiede et al., 2009). Alternatively, the catastrophic oxidative stress may be caused by temporally downstream events such as mitochondrial dysfunction.

Another group of genes, which drew our attention because of a lack of early response, were those encoding heat shock proteins. Expression of these genes was not induced by gentamicin at 3 h, including the HspA group (*Hspa1a, Hspa1b, Hspa5*, and others), the HspB group (*Hspb1, Hspb2*, and others), the HspC group (*Hsp90aa1, Hsp90ab1*, and *Hsp90b1*), and other members in the HspD, HspE, and HspG groups (Table S1). Among Dnaj family members, which are involved in oxidative stress response, only *Dnaja2, Dnajb4*, and some *Dnajc* genes were expressed at slightly higher levels (Table S1). The absence of strong induction of heat shock protein genes suggests that hair cells had not launched a broad stress response at 3 h, and indicates that transcriptional changes in signaling pathways, such as the JNK and the NF-κB pathways, at this time point were not simply caused by dysregulation of general transcription under stress conditions.

#### Genes involved in the initiation and execution of apoptosis were not significantly affected at the early time point

Aminoglycoside antibiotics are thought to induce hair cell death through an intrinsic mitochondrial apoptosis pathway (Forge and Li, 2000). Apoptosis is an active process that requires gene transcription and protein synthesis (Matsui et al., 2002). In contrast to genes involved in upstream pathways, such as the JNK and the NF-κB pathways, which regulate apoptosis and survival, genes involved in apoptosis initiation and execution were not significantly affected in gentamicin-treated hair cells at 3 h post treatment (Figure 5B). Although we observed up-regulation of *Casp3* expression and down-regulation of *Casp2* and *Casp9* expression, the majority of intrinsic mitochondrial apoptosis pathway-related genes remained unchanged, including pro- and anti-apoptosis Bcl2 family members (Figure 5B). The lack of significant induction of apoptosis factors at this relatively early time point is consistent with the critical events timeline of aminoglycoside-induced hair cell death (Matsui et al., 2004). We verified the expression changes of *Bnip1, Casp3* and *Ccar1* by Q-PCR (Figure 5A).

We analyzed the cell-type specific response by comparing gentamicin-induced expression changes in hair cells, with gene expression changes during cell death in fibroblasts, neurons, and kidneys (based on published literature as curated in the IPA data base). Like hair cells, neurons are terminally-differentiated, non-dividing cells, but pathways regulating neuronal cell apoptosis are well characterized. We also emphasized the kidney because nephrotoxicity is another well-known sequela to aminoglycoside antibiotics treatment. These comparisons in IPA revealed that gentamicin-induced gene expression changes in hair cells are significantly different from the dataset derived from fibroblasts (*p* = 0.0000927 and activation z-score −1.81) (activation z-score is a quantitative measure of how similar the hair cell dataset is to the “comparing dataset”: larger positive number indicates there are more genes with expression changes in the same direction in both datasets, while larger negative number means there are more genes with changes in opposite direction between datasets; see Table 1 caption), but similar to datasets of neuronal cell death (*p* = 0.000280 and activation z-score 0.312), and cell death of kidney cells (*p* = 0.00576 and activation z-score 0.664). The observed similarity between aminoglycoside-induced hair cell death and neuronal cell death is not surprising, as hair cells and neurons are related types of terminally differentiated, excitable cells. The similarity between hair cells and kidney cells in response to aminoglycoside-induced stress will be discussed in Section: “Similarity between aminoglycoside ototoxicity and nephrotoxicity” found below.

**Table 1:** **Comparison of the hair cell dataset with known expression changes during cell death in other tissues and cell types**.

**Comparing dataset**	***p*-value**	**Activation z-score**	**# Genes**
Cell death of kidney cells	5.76E-03	0.664	73
Cell death of kidney cell lines	1.09E-03	0.579	67
Neuronal cell death	2.80E-04	0.312	149
Cell death of brain cells	4.10E-04	0.024	63
Cell death of central nervous system cells	6.80E-04	0.015	66
Cell death of fibroblast cell lines	2.71E-04	−0.539	98
Cell death of cerebellar cortex cells	2.77E-05	−0.574	17
Cell death of tumor cell lines	4.70E-06	−0.656	331
Cell death of lymphoma cell lines	2.54E-03	−1.148	46
Cell death of fibroblasts	9.27E-05	−1.81	61

*Selected entries are shown. The p-value, which is calculated using the right-tailed Fisher Exact Test by IPA, is a measure of the likelihood that the association between a set of focus genes in two datasets is due to random chance. Activation z-score is a quantitative measure of how similar the hair cell dataset is to the “comparing dataset”: larger positive number indicates there are more genes with expression changes in the same direction in both datasets, while larger negative number means there are more genes with changes in opposite direction between datasets. # Genes indicates number of overlapping genes in the comparison*.

#### Gentamicin-induced changes in cell cycle and DNA damage repair genes indicate disruption of the postmitotic state

Another group of early response genes whose expression was significantly affected upon exposure to gentamicin includes those involved in cell cycle regulation and DNA damage repair. We verified several genes in this group, including *Ccnb2* (*Cyclin B2*), *Ccne2* (*Cyclin E2*), *Ercc8* (*Csa*), *Rad52, Apex2*, and *Alkbh3* (Figure 6A). Expression changes of genes involved in cell cycle regulation and nucleotide excision repair are shown in Figures 6B,C, respectively. Comparisons between gentamicin-induced gene expression changes and known transcription regulation of related genes during cell cycle or DNA damage repair are summarized in Tables 2, 3, respectively. These data suggest that the postmitotic state of hair cells was disrupted following gentamicin treatment. Loss of control of the postmitotic state and re-entry into the cell cycle preceding neuronal cell apoptosis has been observed (Arendt, 2000), and inhibition of cell cycle re-entry by blocking CDK activity has been shown to protect neurons from apoptosis induced by certain drugs or stress (Kruman, 2004; Golsteyn, 2005). Consistent with these observations in neuronal cells, aberrant cell cycle re-entry, induced by knocking out *Cdkn1a* (*p21*) and *Cdkn2d* (*p19*), leads to hair cell apoptosis (Laine et al., 2007). Inhibition of CDK2 activity also protects hair cells from gentamicin ototoxicity (Tao and Segil, unpublished data), suggesting that the cell cycle machinery plays a role in mediating hair cell death. Our RNA sequencing data showing transcriptional changes to cell cycle-related genes indicate a disruption of the postmitotic state, which supports the idea that loss of cell cycle control may underlie an additional pathway leading to hair cell death after gentamicin treatment.

**Table 2:** **Comparison of the hair cell dataset with known expression changes in datasets from different stages of cell cycle**.

**Comparing dataset**	***p*-value**	**Activation z-score**	**# Genes**
G2 phase	1.08E-06	0.964	69
G2/M phase	1.31E-03	0.507	46
Cell cycle progression	3.96E-07	0.199	237
Mitosis	2.51E-05	−0.075	112
G1 phase	6.42E-04	−0.401	86
G1/S phase transition	1.75E-03	−0.506	46
M phase	2.37E-04	−0.891	55
S phase	1.44E-04	−1.122	63
Interphase	9.91E-08	−1.334	160
Entry into interphase	4.35E-03	−1.523	35

*Selected entries are shown*.

**Table 3:** **Comparison of the hair cell dataset with known expression changes in genes associated with the DNA repair process**.

**Comparing dataset**	***p-*value**	**Activation z-score**	**# Genes**
Repair of DNA	7.05E-06	1.953	66
DNA damage response of cells	6.09E-05	0.343	43
DNA replication	7.48E-05	−0.523	61
Metabolism of DNA	6.37E-04	0.357	90
Excision repair	1.02E-03	2.756	20

Induction of DNA damage repair genes also supports the hypothesis that the postmitotic state is disrupted in hair cells after gentamicin treatment. It is plausible that the induction of DNA repair genes by DNA damage is caused by elevated ROS levels after gentamicin treatment, but our experimental observations do not support this explanation. First, the absence of significant induction of oxidative stress response genes suggests that the ROS production had not reached a level sufficient to induce the change in known response genes. Second, we failed to detect DNA damage by immunostaining against phosphorylated Chk, or by comet assay (Tao and Segil, unpublished data). Third, we did not observe induction of DNA damage repair genes at the transcriptional level after treating the cochlea with the DNA-damaging agent cisplatin (Ng, Rainey and Segil, unpublished data). Based on these observations, we postulate that up-regulated expression of DNA damage repair genes is not triggered by gentamicin-induced DNA damage and ROS production, but rather is more likely to reflect a change in cell cycle status following gentamicin treatment.

### Gene expression changes induced by gentamicin in the non-hair cell population

Hair cells are specifically marked by GFP in Atoh1-GFP transgenic mice (Figure 1A), thus the GFP negative population consists of supporting cells (Deiters' cells, pillar cells and Hensen cells), cells in the greater epithelial ridge, cells in the lesser epithelial ridge, and other cells constituting surrounding tissues (inner phalangeal cells were excluded by FACS, based on their low-level misexpression of GFP, Figures 1A,C). The number of genes whose expression was affected by gentamicin in non-hair cell samples is much smaller than that in hair cells. The PCA results (Figure 1D) also show that the non-hair cell population does not respond to gentamicin uniformly, probably as a result of high heterogeneity. Expression of genes involved in cholesterol synthesis, lipid synthesis, nucleotide metabolism, and other biosynthesis processes, was significantly induced after gentamicin treatment in this population (Table 4). In addition, expression of genes involved in DNA replication and cell cycle was significantly affected (Table 4). The gentamicin-induced gene expression pattern of the non-hair cell population suggests gentamicin-stimulated biosynthesis, and induced cell proliferation in this population.

**Table 4:** **Comparison of non-hair cell dataset with known expression changes in biosynthesis and proliferation datasets**.

**Comparing dataset**	***p*-value**	**Activation z-score**	**# Genes**
Synthesis of sterol	4.76E-11	1.723	16
Synthesis of cholesterol	1.04E-10	1.937	14
Metabolism of cholesterol	3.01E-10	1.937	17
Steroid metabolism	1.38E-05	1.937	20
Steroidogenesis	2.79E-05	1.929	17
Synthesis of terpenoid	3.74E-05	2.075	20
Metabolism of terpenoid	5.42E-05	1.937	21
Metabolism of membrane lipid derivative	6.59E-05	1.66	25
Conversion of acyl-coenzyme A	9.34E-05	1.964	4
Proliferation of cells	3.80E-03	2.161	136

*Selected entries are shown*.

In the presence of Texas-Red conjugated gentamicin (GTTR) in culture medium, hair cells had much higher GTTR fluorescence than other cell types (Figure 1A), suggesting that gentamicin preferentially accumulates in hair cells. However, hair cells and supporting cells form a mosaic linked by tight epithelial junctions and other cell-cell contacts (Gulley and Resse, 1976). Communication between hair cells and supporting cells is continuous as a result of cell-cell contact, and changes in one cell type that result from the gentamicin treatment likely effect physiological change in the other. One such cell-cell signaling pathway significantly affected by gentamicin treatment in non-hair cells is the Notch signaling pathway. *Dll1* and *Jag2* are highly expressed as Notch ligands in hair cells, and *Hes1, Hes5, Hey1*, and *Hey2* are expressed in supporting cells as Notch target genes (Zine and de Ribaupierre, 2002; Doetzlhofer et al., 2009); after gentamicin treatment, expression of *Dll1* and *Jag2* were down-regulated in hair cells, and *Hes1, Hes5, Hey1*, and *Hey2* were also expressed at a lower level in the non-hair cell population (*p* = n.s. for *Hes* and *Hey* genes) (Table S1), indicating disrupted cell-cell signaling between hair cells and supporting cells. This observation supports the idea that the expression profile changes that we observe in the non-hair cell population may be brought about by changes in response to gentamicin in hair cells. However, we could not exclude the possibility that non-supporting cells in the GFP-negative population might be responding to gentamicin treatment directly at the transcriptional level.

### Similarity between aminoglycoside ototoxicity and nephrotoxicity

Nephrotoxicity is another side effect of aminoglycoside antibiotics, and several publications catalog the changes in response to aminoglycoside antibiotics (Oh et al., 2006; Ozaki et al., 2010). A statistical comparison of gentamicin-induced hair cell gene expression changes and transcriptional profiles after gentamicin treatment in kidney identified a significant overlap between gene expression changes in gentamicin-treated hair cells and kidneys (*p* = 8.02E-08). In the dataset obtained from kidney, 211 genes were identified by microarray that were significantly changed after gentamicin treatment (Ozaki et al., 2010), and there were 50 genes in our hair cell dataset that were changed in the same direction as they were in kidney (Table 5).

**Table 5:** **Comparison of gentamicin-induced hair cell gene expression changes with gentamicin-induced expression changes in kidney**.

**Gene symbol**	**Function Annotation**	***p*-value in HC dataset**	**Fold Change in HC dataset**	**Findings in nephrotoxicity**	**Consistent**
Ppp1r10	Phosphatase	5.84E-06	4.524746	Upregulates	Yes
Ddit3	Transcription factor	2.62E-05	2.751878	Upregulates	Yes
Jun	Transcription factor	0.00245	2.732988	Upregulates	Yes
Ccnl1	RNA splicing	4.46E-06	2.482795	Upregulates	Yes
Gnl3	p53 pathway regulation	7E-06	2.127794	Upregulates	Yes
Chka	Phospholipid synthesis	6.28E-07	2.029311	Upregulates	Yes
Ddx39	RNA splicing	4.98E-06	1.991224	Upregulates	Yes
Nop58	Ribosome biogenesis	7.68E-08	1.941254	Upregulates	Yes
Ppp1r15a	Phosphatase	0.009454	1.739694	Upregulates	Yes
Kti12	Unkown	0.008232	1.649551	Upregulates	Yes
Ptbp1	RNA splicing	3.83E-05	1.636352	Upregulates	Yes
Eed	Transcription repressor	8.35E-06	1.601639	Upregulates	Yes
Eif2b2	Translation	3.21E-06	1.572336	Upregulates	Yes
Cul2	Ubiquitin ligase	7.77E-06	1.510931	Upregulates	Yes
Zfp622	Transcription regulator	4.77E-06	1.483148	Upregulates	Yes
Naa15	N-acetyltransferase	0.000102	1.461136	Upregulates	Yes
Smn1	RNA splicing	0.000161	1.45206	Upregulates	Yes
Phax	RNA export	0.006074	1.438425	Upregulates	Yes
Lig4	DNA repair	5.82E-05	1.41918	Upregulates	Yes
Azin1	Metabolism	0.001597	1.388636	Upregulates	Yes
Arf6	Ras superfamily	0.000976	1.388019	Upregulates	Yes
2310044G17Rik	Unkown	0.005972	1.37605	Upregulates	Yes
Eaf1	Transcription factor	0.000222	1.369313	Upregulates	Yes
Cdk13	Transcription elongation	0.000244	1.365288	Upregulates	Yes
Pafah1b1	Inactivates platelet-act. factor	0.000164	1.346669	Upregulates	Yes
Gfer	Growth factor	0.001933	1.324081	Upregulates	Yes
Pelo	Mitosis	0.000148	1.319478	Upregulates	Yes
Tsc22d1	Transcription regulator	0.000157	1.315102	Upregulates	Yes
Ddx50	RNA helicase	0.00147	1.313465	Upregulates	Yes
Gadd45a	MAPK pathway regulator	0.007003	1.30902	Upregulates	Yes
Tars	tRNA synthesis	0.001263	1.292011	Upregulates	Yes
Mdm2	p53 pathway regulation	0.005424	1.28435	Upregulates	Yes
Dnaja2	Chaperone	0.001429	1.282581	Upregulates	Yes
Eif2b4	Translation	0.001361	1.270756	Upregulates	Yes
Sfpq	RNA splicing	0.000749	1.252767	Upregulates	Yes
Cnksr3	Sodium transport	0.00051	1.240931	Upregulates	Yes
Cand1	Ubiquitin ligase	0.003349	1.215329	Upregulates	Yes
Mybbp1a	Transcription regulator	0.000956	1.214954	Upregulates	Yes
Ncl	Ribosome biogenesis	0.00327	1.206809	Upregulates	Yes
Decr2	Metabolism	0.003445	−1.21269	Downregulates	Yes
Asl	Urea cycle	0.000314	−1.27227	Downregulates	Yes
Acsl3	Metabolism	0.000638	−1.27564	Downregulates	Yes
Gstm1	Antioxidant	0.00249	−1.32453	Downregulates	Yes
S100a1	Calcium binding	0.006299	−1.37041	Downregulates	Yes
Abat	Metabolism	0.002967	−1.38497	Downregulates	Yes
Mrpl23	Mitochondrial ribosome	0.003565	−1.38508	Downregulates	Yes
Hacl1	Metabolism	0.002033	−1.43919	Downregulates	Yes
St6galnac3	Glycolipid synthesis	0.005244	−1.46649	Downregulates	Yes
Pcx	Metabolism	0.00279	−1.65712	Downregulates	Yes
Eef1a1	Translation	2.35E-05	−2.0177	Downregulates	Yes
Zfp354a	Unkown	0.001648	1.544937	Downregulates	No
Hyou1	Stress	0.002956	−1.22211	Upregulates	No
Nedd4	Ubiquitin ligase	2.45E-06	−1.27335	Upregulates	No
Ctsd	Protease	0.00174	−1.33383	Upregulates	No
Hspa5	Stress	0.004463	−1.3884	Upregulates	No
Calr	Calcium binding	0.000413	−1.4266	Upregulates	No
Hsp90b1	Chaperone	5.87E-05	−1.56328	Upregulates	No
Egr1	Transcription regulator	0.004467	−1.61013	Upregulates	No
Zfp36l1	Growth factor response	0.001571	−1.65211	Upregulates	No
Pdia4	Protein folding	0.002689	−1.72559	Upregulates	No
Tubb5	Cytoskeleton	0.001836	−2.0123	Upregulates	No
Spp1	Cell-matrix interaction	0.004118	−4.30391	Upregulates	No

We further compared the underlying biological processes and pathways that were affected by gentamicin in kidney and in hair cells, based on the transcriptional changes. We observed similar changes in the expression of genes involved in ribosome biogenesis, RNA processing, and translation in gentamicin-treated kidney and hair cells (Table 5). In addition, up-regulation of expression at the *Jun* gene, and induction of DNA repair gene *Lig4* were observed in both hair cells and kidney. The similarity in transcriptional response to gentamicin between hair cells and kidney cells suggests that there may be some common mechanisms responsible for aminoglycoside ototoxicity and nephrotoxicity, such as translation inhibition.

Despite the aforementioned similarity, the expression of stress response genes differed greatly in gentamicin-treated kidney and hair cells. Expression of oxidative stress response genes and *Hsp* chaperone genes was collectively increased in gentamicin-treated kidney while expression of these genes was not induced in hair cells. The discrepancy might be based on the different time points of sample collection (3 h post-gentamicin treatment for hair cell sample vs. 7 days after treatment for kidney). It was difficult to directly compare the expression of proliferation genes in kidneys with that in hair cells, because the kidney is an organ consisting of heterogeneous cell populations, with both dividing cells and quiescent cells, whereas hair cells are postmitotic.

## Discussion

The causes of hypersensitivity of hair cells to aminoglycoside antibiotics remain unclear, and the prevailing hypothesis of aminoglycoside ototoxicity holds that aminoglycosides induce high levels of ROS leading to apoptotic stimulation. Our purpose in this project was to characterize the hair cell response to aminoglycoside antibiotics at an early time following administration, before the changes associated with apoptosis were apparent. To this end, we analyzed the response of hair cells after 3 h of treatment with gentamicin. As indicated in Figures 2, 3, the JNK pathway and the NF-κB pathway are early responders, with many active components of the pathways showing significant increases. In addition, early changes in cell cycle-related genes, and DNA damage repair genes, signaled the potential disruption of the postmitotic state. In contrast, genes of the apoptosis and stress response pathways remain relatively unchanged at this time point. The transcription profile of hair cells at the early time point suggests that prior to the detection of an ROS response at the transcriptional level, other changes occur that may lead to the stimulation of apoptotic pathways, such as the aforementioned JNK pathway, NF-κB pathway and postmitotic state disruption.

Aminoglycoside-induced ROS production (Hirose et al., 1997), the protective effect of antioxidants (Schacht, 1999), and the NRF2-mediated oxidative stress response (Hoshino et al., 2011), have all been implicated in aminoglycoside ototoxicity. However, we did not observe a significant induction of the transcriptional targets of NRF2 at 3 h in our *in vitro* system. In addition, expression of heat shock chaperons and other stress response genes were not significantly upregulated. These results suggest that, although ROS changes in hair cells are rapid, at 3 h after gentamicin treatment they have not reached a level sufficient to trigger a broad stress response at the transcriptional level. The catastrophic oxidative stress that triggers apoptosis might be caused by continuous ROS build up, or by subsequent events such as mitochondrial dysfunction.

Expression of genes involved in initiation and execution of apoptosis were not significantly induced at this early time point, which is consistent with the established timeline of hair cell apoptosis (Matsui et al., 2004), and in line with the absence of a strong stress response at the transcriptional level. Together, the data suggest that, at this relatively early time point, hair cells are not yet committed to apoptosis. However, we observed expression changes for genes in upstream signaling pathways regulating apoptosis and cell survival. Not surprisingly, comparison of the observed gentamicin-induced changes in hair cells, with those from cell death studies in other cell lines or tissues, revealed a significant similarity in response to stress.

The disruption of the postmitotic state of hair cells is indicated by expression changes of cell cycle-related genes, suggesting that postmitotic state disruption in dying hair cells is another potential mechanism regulating apoptosis in hair cells, similar to what has been reported in postmitiotic neurons. Cell cycle re-entry has been observed in neurons before neuronal cell death, especially in neurodegenerative diseases (Arendt, 2000). In the central nervous system, mature neurons with mutated genes involved in maintaining the postmitotic state (inactivation of *p19^Ink4d^* and *p27^Kip1^*) lose control of the postmitotic state, re-enter the cell cycle, and eventually undergo apoptosis (Zindy et al., 1999). Activation of the cell cycle machinery is considered one of the mechanisms controlling programmed cell death in postmitotic neurons, either through direct regulation of apoptosis pathways, or through checkpoint activation (Kruman, 2004; Golsteyn, 2005). In the cochlea, aberrant cell cycle re-entry leads to hair cell apoptosis (Chen et al., 2003; Laine et al., 2007). Inhibition of CDK activity by pharmaceutical inhibitors or genetic mutations of CDK genes protect hair cells from aminoglycoside ototoxicity (Tao and Segil, unpublished data), suggesting that cell cycle machinery regulates hair cell death. This mechanism is also supported by our expression data. Genes involved in cell cycle progression, G2 phase, and G2/M were significantly affected at the transcriptional level (Table 2), indicating a disruption of the quiescent state of hair cells by gentamicin treatment.

Further evidence supporting the loss of cell cycle control mechanisms includes our observation that hair cells up-regulate the expression of DNA repair genes after gentamicin treatment. Although induction of DNA repair genes by gentamicin-induced DNA damage is a plausible explanation, the lack of strong oxidative stress response at the transcriptional level, failure of DNA damage detection in hair cells after gentamicin treatment (Tao and Segil, unpublished data), and unchanged DNA repair gene expression at the transcriptional level in hair cells after DNA damaging agent treatment (Ng, Rainey and Segil, unpublished data) together suggest that gentamicin-induced DNA repair gene expression is not responding to DNA damage, but may be responding to a form of “replicative stress” induced by the disruption of the postmitotic state, similar to that observed in neurons exiting the postmitotic state (Andrusiak et al., 2012). During final differentiation and withdrawal from cell cycle, many postmitotic cells, such as neurons, which need never replicate their genomes again and thus can resort to repairing only essential genes, down-regulate global genomic DNA repair and rely entirely on transcription-coupled repair to maintain actively transcribed genes (Nouspikel and Hanawalt, 2000). Based on the observation that hair cell homeostasis requires transcription-coupled repair for long-term survival (Nagtegaal et al., 2015), we postulate that DNA repair capacity is regulated during hair cell differentiation and cell cycle withdrawal. Thus, up-regulation of the expression of DNA repair genes may indicate disruption of the quiescent state and cell cycle control.

Taken together, our RNA sequencing data suggests that at 3 h, an early time point after gentamicin treatment, activation of the JNK and NF-κB signaling pathways and the disruption of the quiescent state, indicated by significant transcription changes, precede broad stress response and apoptosis initiation, and that these early responses may regulate aminoglycoside-induced hair cell death.

### Conflict of interest statement

The authors declare that the research was conducted in the absence of any commercial or financial relationships that could be construed as a potential conflict of interest.

## Supplementary material

Table S1 **Gentamicin-induced differential gene expression in hair cells and in the non-hair cell population**.

Table S2 **IPA canonical pathways that were affected by gentamicin in hair cells (pathways discussed in text are highlighted in yellow)**.

Table S3 **Gentamicin-induced expression changes of transcriptional targets of Jun, Nfkb1 and NRF2 (Nfe2l2)**. Red, significantly upregulated target genes; green, significantly downregulated target genes.
